# Monitoring Eastern White Pine Health by Using Field-Measured Foliar Traits and Hyperspectral Data

**DOI:** 10.3390/s24186129

**Published:** 2024-09-23

**Authors:** Sudan Timalsina, Parinaz Rahimzadeh-Bajgiran, Pulakesh Das, José Eduardo Meireles, Rajeev Bhattarai

**Affiliations:** 1 School of Forest Resources, University of Maine, Orono, ME 04469, USA; sudan.timalsina@maine.edu (S.T.); rajeev.bhattarai@maine.edu (R.B.); 2 School of Biology and Ecology, University of Maine, Orono, ME 04469, USA; jose.meireles@maine.edu

**Keywords:** eastern white pine, white pine needle damage, foliar traits, hyperspectral remote sensing, spectral vegetation indices, random forest

## Abstract

Canopy foliar traits serve as crucial indicators of plant health and productivity, forming a vital link between plant conditions and ecosystem dynamics. In this study, the use of hyperspectral data and foliar traits for white pine needle damage (WPND) detection was investigated for the first time. Eastern White Pine (*Pinus strobus* L., EWP), a species of ecological and economic significance in the Northeastern USA, faces a growing threat from WPND. We used field-measured leaf traits and hyperspectral remote sensing data using parametric and non-parametric methods for WPND detection in the green stage. Results indicated that the random forest (RF) model based solely on remotely sensed spectral vegetation indices (SVIs) demonstrated the highest accuracy of nearly 87% and Kappa coefficient (K) of 0.68 for disease classification into asymptomatic and symptomatic classes. The combination of field-measured traits and remote sensing data indicated an overall accuracy of 77% with a Kappa coefficient (K) of 0.46. These findings contribute valuable insights and highlight the potential of both field-derived foliar and remote sensing data for WPND detection in EWP. With an exponential rise in forest pests and pathogens in recent years, remote sensing techniques can prove beneficial for the timely and accurate detection of disease and improved forest management practices.

## 1. Introduction

An exponential increase in the health issues of Eastern White Pine (EWP) (*Pinus strobus* L.) in recent years, in particular white pine needle damage (WPND), a complex of foliar fungal pathogens, is believed to be one of the main drivers of EWP decline [1,2]. WPND-induced premature defoliation significantly alters litterfall and nitrogen dynamics of infested stands, leading to reduced tree growth and vigor [3]. In addition, various other fungal pathogens such as Caliciopsis canker (*Caliciopsis pinea*), Diplodia tip blight or Sphaeropsis blight (*Diplodia sapinea*), and white pine blister rust (*Cronartium ribicola*), pests such as EWP bast scale (*Matsucoccus macrocicatrices Richards*), white pine weevil (*Pissodes strobi*), and others can cause widespread EWP decline through branch flagging, crown thinning, canker formation, and even dieback [4,5]. The abiotic drivers, which are warmer and wetter climates, increase the foliar disease load and enhance the EWP decline [6]. With levels of infestations rising to previously unprecedented records and limited understanding of the underlying mechanisms driving these changes, proper monitoring and management of EWP take priority for the continued survival of EWP that has been a significant contributor to the ecology and economy of the northeastern USA [4,7].

Monitoring for WPND outbreaks using field and aerial detection surveys (ADS) started in 2010 throughout New England in northeastern USA after increased public concern for EWP health [8]. While field and aerial surveying have been immensely useful, due to the brief symptomatic window of WPND, it is difficult to collect comprehensive data on WPND at a landscape scale. The current tools to monitor EWP decline and WPND infections are unable to keep abreast with the rate at which the WPND infestations are increasing, leading to cases being underreported [9,10]. In general, ADS has limitations of being subjective, qualitative, and influenced by weather conditions [11]. Recent advancements in geospatial and remote sensing tools make them a great asset for assessing WPND outbreaks comprehensively. Apart from the potential for the early detection of diseases such as WPND, the availability of remote sensing tools for use in case of an emergency or a global pandemic situation makes them a good alternative to traditional methods [12].

Recent evolution in remote sensing platforms and optical hyperspectral and multispectral sensor capabilities have led to increased use of spectral data, in particular spectral vegetation indices (SVIs), in the detection of pest- and pathogen-induced damages in forested areas [13,14,15]. There have been instances of successful identification of infestation stages using a hyperspectral imaging platform along with machine learning [13] and arguments in favor of using hyperspectral remote sensing for early assessment of tree diseases [16]. Due to the narrowband capturing of information, hyperspectral data offers more comprehensive details compared to multispectral data. Machine learning (ML) algorithms such as random forest (RF) have gained attraction in recent years for regression and classification analysis. RF is a non-parametric ML algorithm consisting of multiple decision trees. It makes classification decisions based on the majority vote from the trees, each trained on a different bootstrap sample of the dataset. RF machine learning algorithm is widely used for predictive modeling, which can handle complex datasets effectively, and helps reduce data dimensionality [17,18]. Such an approach is important for dealing with many variables and is often used in data science due to its high classification accuracy and resistance to overfitting [19].

Smigaj et al. [20] investigated the potential of using multiple sensors, such as hyperspectral and Light Detection and Ranging (LiDAR), to detect disease-induced defoliation with SVIs in pine trees infested with red band needle blight. Rahimzadeh-Bajgiran et al. [11] used Landsat-SVIs and RF modeling for current-year spruce budworm (SBW) defoliation detection where the combination of Normalized Difference Vegetation Index (NDVI), Enhanced Vegetation Index (EVI), and Normalized Difference Moisture Index (NDMI) showed promising results. Sentinel-2 and Hyperion satellite imagery were also successfully used for defoliation detection and classification in spruce trees using SVIs and ML algorithms, including RF and support vector machine (SVM) [21,22]. There are limited studies on detecting WPND using remote sensing data. Meneghini et al. [2] attempted WPND detection and classification using Sentinel-2-derived SVIs and RF modeling and reported 75% accuracy for a two-class severity classification. However, they found detecting light and moderately defoliated stands challenging due to the nature of the WPND, which starts from underneath the canopy. These mandate the exploration of alternative remote sensing techniques, such as detecting potential damage before visual symptoms can be detected (in the green stage). Dash et al. [23] used multi-temporal unmanned aerial vehicle (UAV) imagery to study disease outbreaks in *Pinus radiata* through crown health (density) and needle health (discoloration) monitoring. They applied the RF model for physiological stress classification using spectral indices and found good classification accuracy. They reported that UAV multispectral imagery was useful for identifying physiological stress during the early stages of stress using the red edge and near infra-red bands, wherein NDVI and red-edge NDVI (RENDVI) were important predictor variables. 

Changes in leaf traits such as chlorophyll (Chl), nitrogen (N), and equivalent water thickness (EWT) can serve as a measure of forest health and productivity, as leaf traits are crucial detection tools for monitoring and managing the viability, functioning, and productivity of forest ecosystems [24]. However, there are limited studies available to link foliar traits to forest health assessment [18,25], and there is no literature available to identify key foliar traits for WPND detection in EWP, as well as using hyperspectral data to detect WPND.

The overarching goal of this study was to detect WPND in the green stage and classify it using hyperspectral data integrated with field observation. The objectives were (i) detecting WPND using only field-derived foliar traits and RF algorithm, (ii) detecting WPND using only hyperspectral-derived SVIs variables using RF algorithm, and (iii) detecting WPND using combined field- and hyperspectral-derived variables using RF algorithm.

## 2. Materials and Methods

### 2.1. Study Area

**Figure 1:**
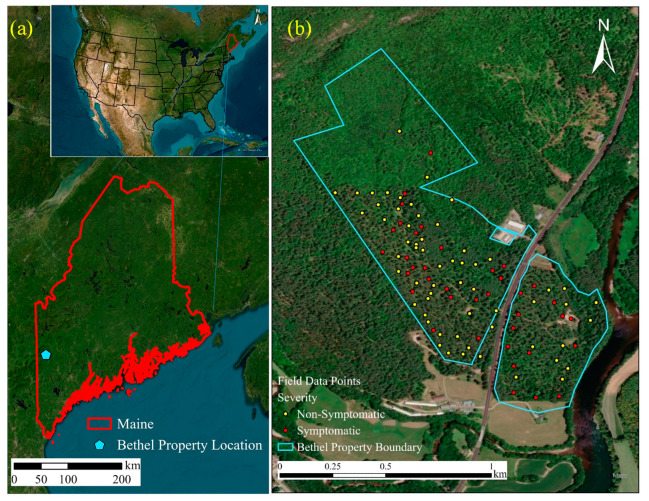
(**a**) The location of the study area in Maine (**b**) the positions of the sampled trees in Bethel (central coordinates: 44.40° N, 70.79° W).

A private property in Bethel, ME, USA (centered at ca. 44.40° N and 70.79° W) was selected as the study area for the project in collaboration with the Maine Forest Service and the US Forest Service. Bethel is a small area in Oxford County, ME, USA, predominantly covered with white pine along with other tree species such as *Acer rubrum*, *Acer saccharum*, *Quercus rubra*, *Quercus alba*, and *Fagus grandifolia*. Before field data collection, a visual assessment of the WPND symptoms was performed to categorize the healthy or unhealthy trees in the study site. The study site was distributed over an area of 71.8 hectares. Figure 1 shows the location of the study area along with the location of the trees sampled in Bethel, Maine.

### 2.2. Field Data Collection

Considering the small symptomatic window of WPND, the EWP needle samples were collected in early summer in their early growing stage or green stage, when the most apparent symptoms of chlorosis (i.e., discolored needles) were unclear. The reasoning behind the field data collection in early summer is to detect the WPND in the green stage. The field data were collected over a period of six weeks from the start of June 2022. Foliage samples were collected from 105 EWP trees, wherein 98 sample trees were selected after data cleaning. The GPS locations of the trees were recorded using a high-precision Trimble GEO 7x and Zephyr external antenna (Sunnyvale, CA, USA), which achieved <1 m positional accuracy after post-processing. 

Through multiple visits, the trees were visually assessed for symptoms of WPND infestation. The healthy-looking trees had green color needles on them, while the ones infested with WPND appeared to have a yellowish color. Early June and mid-June assessments aligned with the pre-defoliation and peak-defoliation periods, respectively. Based on the severity of WPND symptoms, sampled trees were grouped into two groups: symptomatic and asymptomatic. The classification was assisted by input from experts and collaborators at the Maine Forest Service and the US Forest Service. In each plot, two to three branches were collected from each sample tree, depending on the size of the branch. A slingshot was used to shoot down sunlit foliage samples from the exposed upper canopies of the trees. The collected foliage samples from each tree were then wrapped in moist paper towels and stored in separate zip-lock bags to avoid contamination and limit changes in reflectance spectra and biochemical properties of the needles. The zip-lock bags were then stored in a portable cooler with ice packs in a polyethene bag and transported to a laboratory for further analysis [18].

### 2.3. Field Data Processing

#### 2.3.1. Biophysical Measurements

The fresh weight (Fw) for each sample was measured with a digital scale at an accuracy of ~0.001 g. Approximately 3 g of fresh foliage samples were weighed for each sample tree. Similarly, the surface area (SA) of the needles was determined by the AM350 Portable leaf area meter (ADC BioScientific Ltd., Hoddesdon, UK). A correction factor of 2.88, specific for EWP needles, was calculated by using the measurements from AM350 and Winseedle scanner for needles (Regent Instruments Inc., Quebec City, QC, Canada). After applying the correction factor on the needles, the SA data were used to calculate Leaf Mass per Area (LMA) [26]. Chlorophyll fluorescence or fluorescence was also measured with the help of FluorPen (Photon Systems Instruments, Drásov, Czech Republic) to assess the photosynthetic efficiency. 

#### 2.3.2. Hyperspectral Measurements

The remote sensing data (i.e., the hyperspectral signature) for the green samples were recorded within 24 h of field data collection using a Spectra Vista HR 1024 field spectro-radiometer (Spectra Vista Corporation, Poughkeepsie, NY, USA). The leaf-directional hemispherical reflectance from 350 to 2500 nm for each sample was measured by the instrument using the SVC leaf clip with an integrated light source. The spectrometer’s wavelength resolution may vary among detector regions, with a typical spectral resolution of 3–5 nm in the visible (VIS) range and 6–12 nm in the near-infrared (NIR) and shortwave infrared (SWIR) bands. Subsequently, the data were interpolated to achieve a 1 nm resolution [27]. SVC’s supplied Spectralon panel (reflectance ~99%) was used to take white-reference measurements every 10 minutes to minimize the effect of sensor and illumination source drift. The white reference measurements were used to convert the incident radiance of the needles to spectral reflectance (fraction of reflected radiation by spectral band) [28]. The leaf clip that was used to hold the sample had a diameter of about 1 centimeter. To acquire the spectral signature of white pine needles in the green stage, a modified approach was adopted from the standard Spectra Vista Corporation (SVC) leaf measurements protocol by Beth Fallon, originally designed for oak wilt studies [29]. White pine needles from each fascicle were carefully selected to represent the overall health and status of the sample tree. The needles were then placed against a black background included in the SVC leaf clip and carefully stacked to minimize air spaces for a more accurate reflection of the needle’s spectral properties. This step was crucial in reducing external influences on the spectral data.

#### 2.3.3. Chemical Analyses

After spectral signature collection, the Chl, fresh weight, dry weight, leaf area, and biochemical properties of the foliage samples were measured in the laboratory. The chlorophyll content (Chl: µg/cm^2^) was measured using a CCM-300 (Opti-Sciences, Hudson, NH, USA) chlorophyll content meter. The foliar samples were then oven-dried at 65 °C for 72 h to determine the dry weight (D_W_) of the sample. LMA and EWT were calculated using the D_W_, F_W_, and SA of the foliar samples (Equations (1) and (2)) [30,31].

EWT (g/cm^2^) = (F_W_−D_W_)/SA (1)

LMA (g/cm^2^) = (D_W_/SA) (2)

The dried foliar samples were ground into fine powder using a soil grinder, and the powdered sample was sent to the lab for analysis to measure N content. There are two ways to express foliar traits, either area-based concentration (g/m^2^) or mass-based (mg/g or %) [30]. Mass-based N estimate was calculated by multiplying the N (%) value by 10 and area-based estimation for N was done by multiplying the N_mass_ by the LMA.

### 2.4. Methodology

#### 2.4.1. Hyperspectral Data: Spectral Vegetation Indices (SVIs) Calculation

The spectral signature of needle samples was collected, ranging from 400 to 2500 nm in continuous bands (1 nm band gap). Apart from using individual bands, the spectral bands were averaged out by combining 3, 5, 7, and 10 bands. The narrow-band data were used to derive SVIs. A diverse set of narrow-band vegetation indices, such as greenness, chlorophyll, water content, and nitrogen content were generated, as detailed in Table 1. To avoid duplication of similar band combinations, such as (red and NIR), we used statistically independent spectral indices in this study [25].

#### 2.4.2. Differentiating Asymptomatic and Symptomatic Samples Based on Foliar Traits and SVIs

The 98 needle samples were grouped into two categories: asymptomatic and symptomatic (combining light and moderate-severely diseased needles). The foliar traits and SVI values for asymptomatic and symptomatic categories were compared. The t-tests were performed separately for field-measured traits and SVIs to evaluate significant differences between symptomatic and asymptomatic samples.

**Figure 2:**
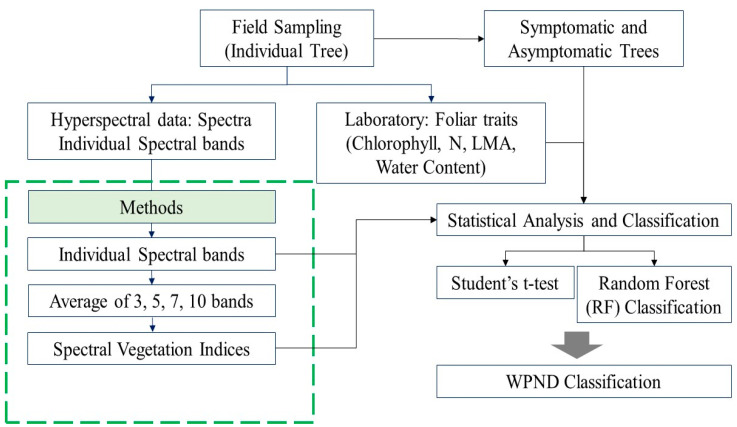
The overall data processing flowchart.

Further, the RF machine learning algorithm [47] was applied to differentiate asymptomatic and symptomatic samples based on spectral reflectance, foliar traits, and SVI values. RF classification was performed in the R programming language. The ‘caret’ and ‘ggplot2’ packages were used. Several RF models were developed based on different input data, such as individual spectral bands, averages of 3, 5, 7, 10, and 22 bands, and SVIs as well as field-measured traits to assess the feasibility of symptomatic and asymptomatic classification. The Boruta function was used to identify important hyperspectral bands for classification and to reduce the data dimension [48]. Further, a correlation coefficient matrix was developed to identify the least correlated bands (correlation < ±0.7), which were then used to build an RF model for WPND classification [17]. Finally, five SVIs (NDVI, GNDVI, REP, NDNI, and NDWI_1240_, Table 2) were selected from the list of SVIs using a threshold correlation coefficient mentioned above [17]. In the case of foliar traits, LMA and EWT were not used in the same combination due to their high correlation (>0.70). NDVI and REP were the indices that had a low correlation and were selected as Chl indicators. NDNI, NI_Wang, and NI_Ferwerda were selected as proxy indices to estimate N, and NDWI_1240_ was selected as an indicator of water content (EWT). The selected variables were then used to build the RF models to check the potential of the machine learning algorithm in classifying the asymptomatic and symptomatic needle samples. Using a random sampling approach, 70% of the reference samples were used in model building, and the remaining 30% were used for model validation. The overall data processing flowchart is shown in Figure 2.

## 3. Results

### 3.1. WPND Detection Using Individual Field-Measured Traits and SVIs

**Figure 3:**
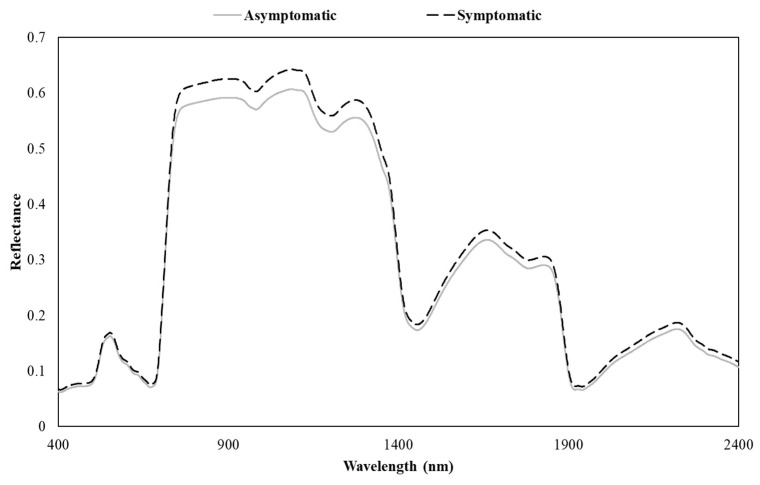
Average spectral signatures of the symptomatic and asymptomatic EWP needles.

Figure 3 depicts the average spectral curve for the symptomatic and asymptomatic trees. The spectra of symptomatic and asymptomatic samples were nearly similar in the red and green regions. However, the highest difference between the symptomatic and asymptomatic samples was observed in the near-infrared region (NIR) compared to other regions, followed by slight differences in the shortwave infrared (SWIR) regions. The spectral signatures of individual samples were used to calculate the SVIs for further analysis. Note that the study area has been experiencing widespread WPND according to input received from the US Forest Service and Maine Forest Service, and therefore, being asymptomatic may not necessarily mean healthy trees. Rather, symptoms were not visible in those samples.

**Figure 4:**
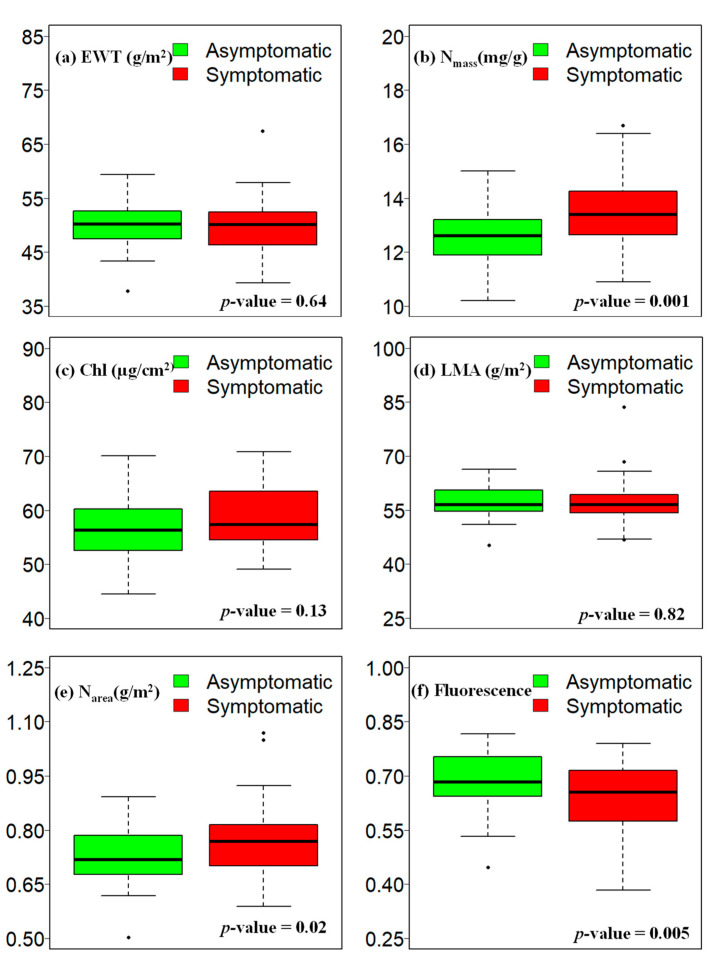
The variations in (**a**) EWT, (**b**) N_mass_, (**c**) Chl, (**d**) LMA, (**e**) N_area_, and (**f**) Fluorescence in asymptomatic and symptomatic EWP samples.

The range and mean differences in trait values were represented through box plots with significance levels (Figure 4). The LMA values for the symptomatic samples ranged from 46 to 84 g/m^2^, while the value range was between 45 and 67 g/m^2^ for the asymptomatic samples. Similarly, mass-based N values ranged from 10.9 to 16.7 mg/g for the symptomatic samples and from 10.2 to 15.0 mg/g for the asymptomatic samples. The EWT values for asymptomatic samples were from 37 to 60 g/m^2^ and from 39 to 68 g/m^2^ for the symptomatic samples. The area-based N values for symptomatic samples ranged between 0.59 and 1.07 g/m^2^, which ranged from 0.50 to 0.89 g/m^2^ for asymptomatic samples. A similar overlap in values was also observed for chlorophyll fluorescence, which varied between 0.38 and 0.79 for the symptomatic samples and between 0.45 and 0.82 for the asymptomatic samples. The Chl, LMA, and EWT did not show significant differences between asymptomatic and symptomatic samples. However, the area, mass-based N, and fluorescence are the only traits that showed significant differences (*p* < 0.05) between asymptomatic and symptomatic samples. The N content indicated a negative correlation with tree health, wherein a higher N concentration was recorded in symptomatic samples.

**Figure 5:**
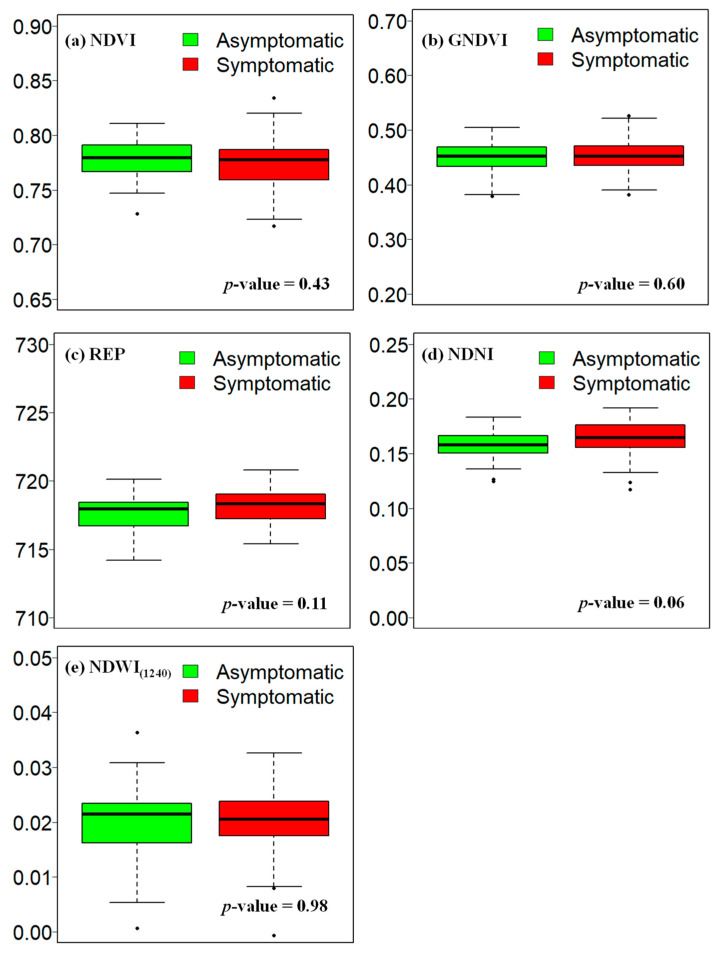
The variations of (**a**) NDVI, (**b**) GNDVI, (**c**) REP, (**d**) NDNI, and (**e**) NDWI_1240_ in asymptomatic and symptomatic EWP samples.

The difference in SVIs values between asymptomatic and symptomatic samples is shown using the box plots (Figure 5). The SVIs selected based on the correlation matrix were studied in this case (Table S1). The results indicated no statistically significant (*p* < 0.05) mean difference between the asymptomatic and symptomatic samples. However, the most notable differences were observed for REP and NDNI, with the *p*-value for NDNI at 0.06 (*p* < 0.1) approaching statistical significance. 

### 3.2. WPND Detection and Classification Using Random Forest (RF)

Although individual and averaged out spectral bands (i.e., variables) were used for WPND classification using the RF model, the use of these bands for WPND classification did not yield promising results as compared to the RF model with the SVIs, and therefore, they were excluded from further analysis. 

The RF models were developed using three combinations: field-measured traits only, remotely sensed SVIs only and both field-measured traits and remotely sensed SVIs together. The overall accuracies and Kappa coefficient (K) of the RF models in classifying WPND are presented in Table 2. Using only field-measured traits, the highest classification accuracy (overall accuracy: 70% and K: 0.38) was observed for the model that included four traits of EWT, Chl, N_mass_, and fluorescence. In comparison, using only remotely sensed SVIs and the integration of remotely sensed SVIs with field-measured traits significantly increased the modeling accuracy. The best-performing RF model based on only remotely sensed SVIs was the one that used the SVIs derived from the correlation matrix (NDVI, NDNI, NDWI_1240_, and REP) with an overall accuracy of 80.00% and K value of 0.52. These SVIs represent at least one of the foliar traits (Chl, N, or EWT). For instance, NDWI_1240_ represents EWT, NDNI represents N content, and NDVI, GNDVI, and REP represent Chl content (Table 2). Adding GNDVI to the best-performing SVIs model increases the model accuracy to 86.7% with a K value of 0.68 despite the correlation between GNDVI and NDVI. The integration of field-measured traits (EWT, Chl, fluorescence, and N_mass_) and SVIs (NDVI, NDNI, NDWI_1240_, and REP) performed well, reaching an accuracy as high as 77% and the K value of 0.46 but still underperformed the model based on only SVIs. Replacing N_area_ with fluorescence did not change the overall accuracy, but the K was slightly higher at 0.31. Table 3 shows the confusion matrix for the best-performing combination in the RF model based on field-measured foliar traits and remotely sensed SVIs, respectively. 

**Figure 6:**
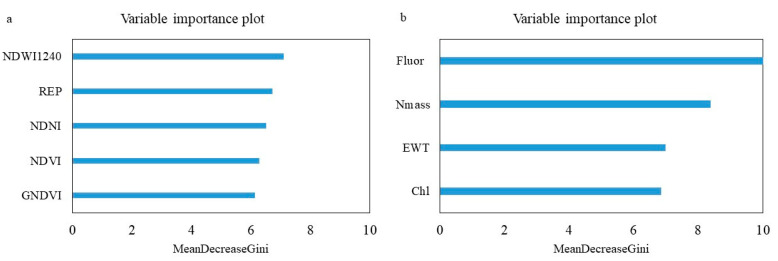
The Variable Importance Plot (VIP) for (**a**) the best-performing RF model using remotely sensed SVIs and (**b**) the best RF model using field-measured traits.

Figure 6 shows the variable importance plot for (a) the best-performing RF model using remotely sensed SVIs and (b) the RF model using field-measured foliar traits. In the RF model based on only SVIs, out of the 5 top variables, the NDWI_1240_ was identified as the most important, followed by NDVI, NDVI-green, NDNI, and REP. However, the relative importance of the variables is close to each other. For the field-measured traits, EWT was identified as the most important, followed by N_area_, N_mass_, and Chl. The relative importance of field-measured traits is similar. In both models, water, N, and Chl contents were the most important variables for WPND classification (represented as EWT, N, and Chl in the field-measured model and NDWI_1240_, NDNI, and a combination of NDVI, NDVI-green, and REP, respectively). Water content (represented as EWT in the field-measured model and NDWI_1240_ in the remotely sensed SVIs model) was identified as the most important variable in classifying the asymptomatic and symptomatic samples.

## 4. Discussion

This study used field-sampled needles from a known WPND-affected site, Bethel, ME, USA, to measure foliar traits and spectral data. The overarching goal of the study was to detect WPND in the green stage when infected needles do not exhibit any visual symptoms. We found that the average spectral reflectance values for the symptomatic samples were higher compared to the asymptomatic samples for most parts of the spectrum. Observable differences were present between the two classes in the NIR and SWIR regions. Most of the indices that exhibited good performance for classification in the RF model were also concentrated within this region. Abdullah et al. [49] compared the spectral signature of healthy and unhealthy leaves of Norway spruce species. They observed a higher reflectance for the unhealthy samples in the visible region, while a higher reflectance for healthy samples in the NIR region, and nearly identical reflectance in the SWIR region. The comparison of foliar traits by Abdullah et al. [49] indicated higher Chl and N content in the healthy samples compared to the unhealthy samples. However, in this study, the Chl and N content values were estimated higher in the symptomatic samples. McIntire et al. [3] studied the N content changes due to WPND in the New England area. They observed a similar trend in N content (i.e., nigher N content in unhealthy samples than healthy samples) and reported that the WPND causes partial N resorption to compensate for the premature needle cast in infested trees. They reported a lower N content (0.8% to 1.3%) in needles during June and July compared to this study where the N content was slightly higher (1.0% to 1.7%). The only field-measured trait that was significantly lower in symptomatic samples was Chl. fluorescence. 

The comparison of foliar traits in the asymptomatic and symptomatic samples indicated very low and insignificant differences except for N in the early growing season or green stage. Similarly, the SVI values indicated insignificant differences between asymptomatic and symptomatic. In contrast, the RF ML model, which included a combination of multiple variables (SVIs and traits) classified the asymptomatic and symptomatic samples well. It could be inferred that although there was an insignificant difference in SVIs between asymptomatic and symptomatic samples, the RF ML model could capture the complex relation between foliar traits, spectral indices, and health classes. The RF model with multiple determinant variables was useful in classifying the asymptomatic and symptomatic samples. Rahimzadeh-Bajgiran et al. [11] used Landsat data-derived SVIs for spruce budworm classification. They reported a higher accuracy for the model, which included multiple SVIs (NDMI, EVI and NDVI) than models that used a single SVI. Bhattarai et al. [21] employed Sentinel-2 data-derived SVIs for spruce budworm defoliation mapping. They reported improvement in the classification with multiple indices and observed a higher accuracy using the RF model than the SVM. The comparison of variable combinations indicated that the remote sensing variables performed better than the field-measured traits in classifying the WPND. Although there is no literature available to be directly compared with this study, the vegetation water indices and chlorophyll indices such as NDWI and NDVI, as well as red-edge indices, were found important for differentiating WPND severity in general [2,11,21]. Syifa et al. [50] used drone-captured remote sensing images and applied an artificial neural network (ANN) and SVM to distinguish between healthy trees and unhealthy (wilt disease) pines. Their study reported high classification accuracy with ML models and observed a higher modeling accuracy with SVM (94.13% and 86.59%) compared to ANN (87.43% and 79.33%). Their study indicated that the remote sensing variables could model the foliar traits, wherein the NDWI_1240_ was identified as the most important variable and REP as the least important variable. As for the leaf traits, EWT was identified as the most important, and Chl was classified as the least important. Past studies mostly compared the spectral data collected for different phases of disease infection and/or samples with visually identifiable differences [51,52]. In contrast, the current study employed spectra from the green stage in the early growing season, wherein no significant difference was visible in the needles. 

This study recommends that damage detection of WPND at the green stage is in the early stages of research and needs further exploration. According to input received from the US Forest Service and Maine Forest Service, most of the EWP trees in the Bethel are experiencing WPND. Therefore, future studies may include additional reference data points from different healthy and unhealthy sites in Maine and surrounding states for comparison and to develop more robust classification models. Future studies may include fine-resolution hyperspectral data, such as drone hyperspectral imageries, for WPND assessments at a local scale. However, the SVIs identified in the current study can also be applied to broadband remote sensing satellite images, such as Sentinel-2 with red-edge spectral bands for landscape level WNPD assessment. 

## 5. Conclusions

WPND impact studies using satellite remote sensing are challenging as the top canopy often remains unaltered, whereas the lower canopy experiences higher damage, yellowing, and premature needle defoliation. This work established a foundation for further application remote sensing EWP health monitoring and early detection of WPND and showed that spectral data could be capable of capturing more information compared to what can be obtained from field-based trait measurement. Non-parametric machine learning models performed well due to the non-linear relationship which exists between the SVIs/traits and WPND. The study highlights that the commonly measured traits may not be optimal for the detection of WPND. Overall, the results from the spectral analysis presented in this work are promising and may help identify other field-measured traits that could be potentially better indicators of EWP health. Moreover, the developed approach and best SVIs identified in this study can be applied or tested with suitable satellite data, such as Landsat and Sentinel-2, for landscape-level WPND impact studies.

## Figures and Tables

**Table 1:** List of SVIs used in the study.

Acronyms (SVIs)	Formula	Reference
**Greenness/Chlorophyll content**		
NDVI	(R_842_−R_665_)/(R_842_+R_665_)	[32]
EVI	2.5×(R_865_−R_665_)/(1+R_865_+6×R_665_−7.5×R_490_)	[33]
GNDVI	(R_750_−R_550_)/(R_750_+R_550_)	[34]
IRECI	(R_783_−R_665_)×(R_740_/R_705_)	[35]
MTCI	(R_740_−R_705_)/(R_705_−R_665_)	[36]
RENDVI	(R_750_−R_705_)/(R_750_+R_705_)	[37]
REP	705+35×(([ R_783_+R_665_]/2)−R_705_)/[R_740_−R_705_]	[38]
**Water content**		
NDWI_1640_	(R_858_−R_1640_)/(R_858_+R_1640_)	[39]
NDII	(R_819_−R_1649_)/(R_819_+R_1649_)	[40]
NDWI_1240_	(R_980_−R_1240_)/(R_980_+R_1240_)	[41]
NMDI	[R_860_−(R_1640_−R_2130_)]/[R_860_+(R_1640_−R_2130_)]	[42]
**Nitrogen content**		
NDNI	[log_10_(1/R_1510_)−log_10_(1/R_1680_)]/[log_10_(1/R_1510_)+log_10_(1/R_1680_)]	[43]
NI_Tian	(R_705_)/(R_717_+R_491_)	[44]
NI_Ferwerda	(R_693_−R_1770_)/(R_693_+R_1770_)	[45]
NI_Wang	[R_924_−R_703_+2×R_423_)]/[R_924_+R_703_−2×R_423_)]	[46]

NDVI: Normalized Difference Vegetation Index, EVI: Enhanced Vegetation Index, GNDVI: Green NDVI, IRECI: Inverted Red Edge Chlorophyll Index, MTCI: MERIS Terrestrial Chlorophyll Index, RENDVI: Red-edge NDVI, REP: Red-edge Position, NDWI: Normalized Difference Water Index, NDII: Normalized Difference Infrared Index, NMDI: Normalized Multi-band Drought Index, NDNI: Normalized Difference Nitrogen Index, and NI: Nitrogen Index.

**Table 2:** Results of RF classification of symptomatic and asymptomatic samples.

Variables (Only Traits)	Accuracy (%)	Kappa
EWT+Chl+N_mass_+Fluorescence	70.0	0.38
N_mass_+Fluorescence	66.7	0.33
EWT+N_mass_+Fluorescence	66.7	0.30
EWT+Chl+N_area_	66.7	0.21
LMA+Chl+N_area_+Fluorescence	63.3	0.25
EWT+Chl+N_area_+Fluorescence	63.3	0.25
LMA+Chl+N_mass_+Fluorescence	60.0	0.20
EWT+Chl+N_mass_	60.0	0.10
**Variables (Only Spectral Vegetation Indices)**		
NDNI+GNDVI+NDVI+REP+NDWI_1240_	86.7	0.68
NDVI+NDNI+NDWI_1240_+REP	80.0	0.52
NDNI+NDVI+NDWI_1240_	73.3	0.40
NDNI+NI_Wang+NI_Ferwerda	66.7	0.15
**Variables (Traits and Spectral Vegetation Indices)**		
NDVI+NDNI+NDWI_1240_+REP+N_mass_+Chl+EWT	76.7	0.46
NDVI+NDNI+NDWI_1240_+N_area_+Chl+EWT	73.3	0.44
NDVI+NDNI+NDWI_1240_+N_mass_+Chl+EWT	73.3	0.43
NDNI+NI_Wang+NI_Ferwerda+NDWI_1240_+N_mass_+Chl+EWT+Fluorescence	70.0	0.42
NDVI+NDNI+NDWI_1240_+REP+N_area_+Chl+EWT	70.0	0.38
NDVI+NDNI+REP+N_area_+Chl+EWT	70.0	0.38
NDNI+NI_Wang+NI_Ferwerda+NDWI_1240_+N_area_+Chl+EWT	70.0	0.31
NDWI_1240_+N_mass_+Chl+EWT+Fluorescence	66.7	0.30
NDWI_1240_+N_area_+Chl+EWT+Fluorescence	66.7	0.30
NDWI_1240_+N_mass_+Chl+EWT	66.7	0.25
NDWI_1240_+N_area_+Chl+EWT	66.7	0.21
NDNI+NI_Wang+NI_Ferwerda+N_mass_+Chl+EWT	63.3	0.29
NDNI+NI_Wang+NI_Ferwerda+NDWI_1240_+N_mass_+Chl+EWT	63.3	0.20

**Table 3:** The confusion matrix for the most accurate RF models based on remotely sensed SVIs and field-measured traits.

	**Remotely Sensed SVIs**			
**Class**	**Asymptomatic**	**Symptomatic**	**Row Sum**	**User** **Accuracy (%)**
Asymptomatic	7	2	9	77.8
Symptomatic	2	19	21	90.5
Column sum	9	21		
Producer Accuracy (%)	77.8	90.5	Overall accuracy (%): 86.7	
			Kappa: 0.68	
	**Field-Measured Traits**			
**Class**	**Asymptomatic**	**Symptomatic**	**Row Sum**	**User** **Accuracy (%)**
Asymptomatic	5	5	10	50.0
Symptomatic	4	16	20	80.0
Column sum	9	21		
Producer accuracy (%)	55.6	76.2	Overall accuracy (%): 70.0	
			Kappa: 0.38	

## Data Availability

The data presented in this study are available on request from the corresponding author.

## Supplementary Materials

The following supporting information can be downloaded at: https://www.mdpi.com/article/10.3390/s24186129/s1, Table S1. The Correlation matrix for the SVIs: Variable Selection & Reduction (Yellow: SVIs with the highest correlations; Green: selected SVIs for further analysis).

## References

[1] Broders K., Munck I., Wyka S., Iriarte G., Beaudoin E. (2015). Characterization of Fungal Pathogens Associated with White Pine Needle Damage (WPND) in Northeastern North America. Forests.

[2] Meneghini A., Rahimzadeh-Bajgiran P., Livingston W., Weiskittel A. (2022). Detecting White Pine Needle Damage through Satellite Remote Sensing. Can. J. For. Res..

[3] McIntire C.D., Munck I.A., Vadeboncoeur M.A., Livingston W.H., Asbjornsen H. (2018). Impacts of White Pine Needle Damage on Seasonal Litterfall Dynamics and Wood Growth of Eastern White Pine (*Pinus Strobus*) in Northern New England. For. Ecol. Manag..

[4] Costanza K.K.L., Whitney T.D., McIntire C.D., Livingston W.H., Gandhi K.J.K. (2018). A Synthesis of Emerging Health Issues of Eastern White Pine (*Pinus Strobus*) in Eastern North America. For. Ecol. Manag..

[5] Munck I.A., Yamasaki M., Janelle J. (2023). Silvicultural treatments improve pest and disease conditions of white pine (*Pinus strobus*) residual trees and regeneration. Front. For. Glob. Change.

[6] Wyka S.A., Munck I.A., Brazee N.J., Broders K.D. (2018). Response of eastern white pine and associated foliar, blister rust, canker and root rot pathogens to climate change. For. Ecol. Manag..

[7] Wyka S.A., Smith C., Munck I.A., Rock B.N., Ziniti B.L., Broders K. (2017). Emergence of White Pine Needle Damage in the Northeastern United States Is Associated with Changes in Pathogen Pressure in Response to Climate Change. Glob. Change Biol..

[8] Munck I., Burns B., Ostrofsky W., Lombard K., Weimer J. (2012). Eastern White Pine Needle Damage Survey, 2011. Maine, New Hampshire, and Vermont.

[9] Bergdahl A., Munck I.A., Lilja R., Cancelliere J., Cole R., Halman J., Keleher N., Lombard K., Weimer J., Ricard P., Potter K.M., Conkling B.L. (2022). Monitoring Eastern White Pine Decline and Its Causes in New England and New York Through Enhanced Survey Methods. Forest Health Monitoring: National Status, Trends, and Analysis 2021.

[10] Potter K.M., Conkling B.L. (2022). Forest Health Monitoring: National Status, Trends, and Analysis 2021.

[11] Rahimzadeh-Bajgiran P., Weiskittel A.R., Kneeshaw D., MacLean D.A. (2018). Detection of Annual Spruce Budworm Defoliation and Severity Classification Using Landsat Imagery. Forests.

[12] Hanavan R.P., Kamoske A.G., Schaaf A.N., Eager T., Fisk H., Ellenwood J., Warren K., Asaro C., Vanderbilt B., Hutten K. (2022). Supplementing the Forest Health National Aerial Survey Program with Remote Sensing during the COVID-19 Pandemic: Lessons Learned from a Collaborative Approach. J. For..

[13] Haagsma M., Page G.F.M., Johnson J.S., Still C., Waring K.M., Sniezko R.A., Selker J.S. (2020). Using Hyperspectral Imagery to Detect an Invasive Fungal Pathogen and Symptom Severity in Pinus Strobiformis Seedlings of Different Genotypes. Remote Sens..

[14] Hall R.J., Castilla G., White J.C., Cooke B.J., Skakun R.S. (2016). Remote Sensing of Forest Pest Damage: A Review and Lessons Learned from a Canadian Perspective. Can. Entomol..

[15] Lausch A., Erasmi S., King D.J., Magdon P., Heurich M. (2017). Understanding Forest Health with Remote Sensing-Part II-A Review of Approaches and Data Models. Remote Sens..

[16] Niemann K.O., Quinn G., Stephen R., Visintini F., Parton D. (2015). Hyperspectral Remote Sensing of Mountain Pine Beetle with an Emphasis on Previsual Assessment. Can. J. Remote Sens..

[17] Bhattarai R., Rahimzadeh-Bajgiran P., Weiskittel A. (2022). Multi-Source Mapping of Forest Susceptibility to Spruce Budworm Defoliation Based on Stand Age and Composition across a Complex Landscape in Maine, USA. Can. J. Remote Sens..

[18] Bhattarai R., Rahimzadeh-Bajgiran P., Mech A. (2023). Estimating Nutritive, Non-Nutritive and Defense Foliar Traits in Spruce-Fir Stands Using Remote Sensing and Site Data. For. Ecol. Manag..

[19] Belgiu M., Drăgu L. (2016). Random Forest in Remote Sensing: A Review of Applications and Future Directions. ISPRS J. Photogramm. Remote Sens..

[20] Smigaj M., Gaulton R., Suárez J.C., Barr S.L. (2019). Combined use of spectral and structural characteristics for improved red band needle blight detection in pine plantation stands. For. Ecol. Manag..

[21] Bhattarai R., Rahimzadeh-Bajgiran P., Weiskittel A., MacLean D.A. (2020). Sentinel-2 Based Prediction of Spruce Budworm Defoliation Using Red-Edge Spectral Vegetation Indices. Remote Sens. Lett..

[22] Donovan S.D., MacLean D.A., Zhang Y., Lavigne M.B., Kershaw J.A. (2021). Evaluating Annual Spruce Budworm Defoliation Using Change Detection of Vegetation Indices Calculated from Satellite Hyperspectral Imagery. Remote Sens. Environ..

[23] Dash J.P., Watt M.S., Pearse G.D., Heaphy M., Dungey H.S. (2017). Assessing very high resolution UAV imagery for monitoring forest health during a simulated disease outbreak. ISPRS J. Photogramm. Remote Sens..

[24] Wright I.J., Reich P.B., Westoby M., Ackerly D.D., Baruch Z., Bongers F., Cavender-Bares J., Chapin T., Cornelissen J.H.C., Diemer M. (2004). The Worldwide Leaf Economics Spectrum. Nature.

[25] Abdullah H., Skidmore A.K., Darvishzadeh R., Heurich M. (2019). Sentinel-2 Accurately Maps Green-Attack Stage of European Spruce Bark Beetle (Ips Typographus, L.) Compared with Landsat-8. Remote Sens. Ecol. Conserv..

[26] Gara T.W., Rahimzadeh-Bajgiran P., Darvishzadeh R. (2021). Forest Leaf Mass per Area (LMA) through the Eye of Optical Remote Sensing: A Review and Future Outlook. Remote Sens..

[27] Burnett A.C., Anderson J., Davidson K.J., Ely K.S., Lamour J., Li Q., Morrison B.D., Yang D., Rogers A., Serbin S.P. (2021). A Best-Practice Guide to Predicting Plant Traits from Leaf-Level Hyperspectral Data Using Partial Least Squares Regression. J. Exp. Bot..

[28] Holzman M., Rivas R., Bayala M., Pasapera J. (2021). Measuring Land Surface Temperature, near-Infrared and Short-Wave Infrared Reflectance for Estimation of Water Availability in Vegetation. MethodsX.

[29] SVC Leaf Measurements-Cavender-Bares Lab. https://cavender-bares-lab.github.io/Data-management-lab/protocols/svc_leaf_measurements/#svc-hr1024i-software-for-data-collection.

[30] Gara T.W., Rahimzadeh-Bajgiran P., Weiskittel A. (2022). Determination of Foliar Traits in an Ecologically Distinct Conifer Species in Maine Using Sentinel-2 Imagery and Site Variables: Assessing the Effect of Leaf Trait Expression and Upscaling Approach on Prediction Accuracy. ISPRS J. Photogramm. Remote Sens..

[31] Danson F.M., Steven M.D., Malthus T.J., Clark J.A. (1992). High-spectral resolution data for determining leaf water content. Int. J. Remote Sens..

[32] Rouse J.W., Haas R.H., Schell J.A., Deering D.W., Harlan J.C. Monitoring the Vernal Advancement and Retrogradation (Green Wave Effect) of Natural Vegetation. NASA/GSFC Type III Final Rep. Greenbelt Md 1974, 371.

[33] Huete A., Didan K., Miura T., Rodriguez E.P., Gao X., Ferreira L.G. (2002). Overview of the Radiometric and Biophysical Performance of the MODIS Vegetation Indices. Remote Sens. Environ..

[34] Gitelson A.A., Merzlyak M.N., Lichtenthaler H.K. (1996). Detection of Red Edge Position and Chlorophyll Content by Reflectance Measurements Near 700 Nm. J. Plant Physiol..

[35] Clevers J., De Jong S.M., Epema G.F., Addink E.A. (2000). MERIS and the Red-Edge Index. Second EARSeL Workshop on Imaging Spectroscopy, Enschede. https://www.researchgate.net/profile/Jgpw-Clevers/publication/228608329_MERIS_and_the_red-edge_index/links/0fcfd506d6ab306e69000000/MERIS-and-the-red-edge-index.pdf.

[36] Dash J., Curran P. (2004). The MERIS terrestrial chlorophyll index. Int. J. Remote Sens..

[37] Gitelson A., Merzlyak M.N. (1994). Spectral Reflectance Changes Associated with Autumn Senescence of *Aesculus hippocastanum* L. and *Acer platanoides* L. Leaves. Spectral Features and Relation to Chlorophyll Estimation. J. Plant Physiol..

[38] Guyot G., Baret F. Utilisation de la haute resolution spectrale pour suivre l’etat des couverts vegetaux. Proceedings of the Spectral Signatures of Objects in Remote Sensing.

[39] Chen D., Huang J., Jackson T.J. (2005). Vegetation Water Content Estimation for Corn and Soybeans Using Spectral Indices Derived from MODIS Near- and Short-Wave Infrared Bands. Remote Sens. Environ..

[40] Klemas V., Smart R. (1983). The Influence of Soil Salinity, Growth Form, and Leaf Moisture on-the Spectral Radiance of Spartina alterniflora Canopies. Photogramm. Eng. Remote Sens..

[41] Gao B.-C. (1996). NDWI-A Normalized Difference Water Index for Remote Sensing of Vegetation Liquid Water from Space. Remote Sens. Environ..

[42] Wang L., Qu J.J. (2007). NMDI: A Normalized Multi-band Drought Index for Monitoring Soil and Vegetation Moisture with Satellite Remote Sensing. Geophys. Res. Lett..

[43] Serrano L., Penuelas J., Ustin S.L. (2002). Remote Sensing of Nitrogen and Lignin in Mediterranean Vegetation from AVIRIS Data: Decomposing Biochemical from Structural Signals. Remote Sens. Environ..

[44] Tian Y.C., Yao X., Yang J., Cao W.X., Hannaway D.B., Zhu Y. (2011). Assessing Newly Developed and Published Vegetation Indices for Estimating Rice Leaf Nitrogen Concentration with Ground- and Space-Based Hyperspectral Reflectance. Field Crops Res..

[45] Ferwerda J.G., Skidmore A.K., Mutanga O. (2005). Nitrogen Detection with Hyperspectral Normalized Ratio Indices across Multiple Plant Species. Int. J. Remote Sens..

[46] Wang W., Yao X., Yao X.F., Tian Y.C., Liu X.J., Ni J., Cao W.X., Zhu Y. (2012). Estimating Leaf Nitrogen Concentration with Three-Band Vegetation Indices in Rice and Wheat. Field Crops Res..

[47] Breiman L. (2001). Random Forests. Mach. Learn..

[48] Kursa M.B., Rudnicki W.R. (2010). Feature Selection with the Boruta Package. J. Stat. Softw..

[49] Abdullah H., Darvishzadeh R., Skidmore A.K., Groen T.A., Heurich M. (2018). European Spruce Bark Beetle (*Ips Typographus*, L.) Green Attack Affects Foliar Reflectance and Biochemical Properties. Int. J. Appl. Earth Obs. Geoinf..

[50] Syifa M., Park S.J., Lee C.W. (2020). Detection of the Pine Wilt Disease Tree Candidates for Drone Remote Sensing Using Artificial Intelligence Techniques. Engineering.

[51] Asner G.P., Martin R.E., Keith L.M., Heller W.P., Hughes M.A., Vaughn N.R., Hughes R.F., Balzotti C. (2018). A Spectral Mapping Signature for the Rapid Ohia Death (ROD) Pathogen in Hawaiian Forests. Remote Sens..

[52] Scholten R.C., Hill J., Werner W., Buddenbaum H., Dash J.P., Gomez Gallego M., Rolando C.A., Pearse G.D., Hartley R., Estarija H.J. (2019). Hyperspectral VNIR-Spectroscopy and Imagery as a Tool for Monitoring Herbicide Damage in Wilding Conifers. Biol. Invasions.

