# Supplementary material for: Monitoring Eastern White Pine Health by Using Field-Measured Foliar Traits and Hyperspectral Data
Source: Sensors (Basel). 2024 Sep 23;24(18):6129. doi: 10.3390/s24186129 (PMC11435692; doi:10.3390/s24186129)
Supplement: Supplementary file 1 [file sensors-24-06129-s001.zip › sensors-3176636-supplementary.pdf]

Table S1. The Correlation matrix for the SVIs: Variable Selection & Reduction (Yellow: SVIs with the highest correlations; Green: selected SVIs for further analysis)

	NDNI	NI_Tian	NI_Wang	NI_Ferwerda	GNDVI	mNDVI705	NDVI	IRECI	NDII	$NDWI_{1640}$	NMDI	MTCI	EVI8	EVI7	REP	$NDWI_{1240}$
**NDNI**	1.00	-0.37	0.26	-0.30	0.52	0.56	0.54	0.91	0.64	0.66	0.59	0.49	0.95	0.94	0.57	0.40
**NI_Tian**	-0.37	1.00	-0.89	0.70	-0.84	-0.92	-0.47	-0.61	-0.43	-0.44	-0.32	-0.97	-0.40	-0.40	-0.94	-0.39
**NI_Wang**	0.26	-0.89	1.00	-0.41	0.57	0.69	0.08	0.43	0.25	0.26	0.30	0.82	0.30	0.30	0.79	0.27
**NI_Ferwerda**	-0.30	0.70	-0.41	1.00	-0.86	-0.79	-0.74	-0.47	-0.13	-0.15	0.09	-0.71	-0.22	-0.21	-0.71	-0.01
**GNDVI**	0.52	-0.84	0.57	-0.86	1.00	0.96	0.80	0.73	0.52	0.54	0.33	0.90	0.49	0.48	0.91	0.35
**mNDVI705**	0.56	-0.92	0.69	-0.79	0.96	1.00	0.75	0.79	0.62	0.64	0.45	0.97	0.57	0.57	0.97	0.49
**NDVI**	0.54	-0.47	0.08	-0.74	0.80	0.75	1.00	0.69	0.64	0.65	0.31	0.57	0.52	0.52	0.60	0.42
**IRECI**	0.91	-0.61	0.43	-0.47	0.73	0.79	0.69	1.00	0.78	0.80	0.71	0.73	0.94	0.94	0.77	0.61
**NDII**	0.64	-0.43	0.25	-0.13	0.52	0.62	0.64	0.78	1.00	1.00	0.84	0.53	0.75	0.75	0.57	0.85
**$NDWI_{1640}$**	0.66	-0.44	0.26	-0.15	0.54	0.64	0.65	0.80	1.00	1.00	0.84	0.54	0.76	0.76	0.59	0.83
**NMDI**	0.59	-0.32	0.30	0.09	0.33	0.45	0.31	0.71	0.84	0.84	1.00	0.43	0.73	0.74	0.47	0.82
**MTCI**	0.49	-0.97	0.82	-0.71	0.90	0.97	0.57	0.73	0.53	0.54	0.43	1.00	0.51	0.51	0.98	0.45
**EVI8**	0.95	-0.40	0.30	-0.22	0.49	0.57	0.52	0.94	0.75	0.76	0.73	0.51	1.00	1.00	0.58	0.58
**EVI7**	0.94	-0.40	0.30	-0.21	0.48	0.57	0.52	0.94	0.75	0.76	0.74	0.51	1.00	1.00	0.57	0.60
**REP**	0.57	-0.94	0.79	-0.71	0.91	0.97	0.60	0.77	0.57	0.59	0.47	0.98	0.58	0.57	1.00	0.44
**$NDWI_{1240}$**	0.40	-0.39	0.27	-0.01	0.35	0.49	0.42	0.61	0.85	0.83	0.82	0.45	0.58	0.60	0.44	1.00
